# Qualifications for Parenthood

**Published:** 1913-01

**Authors:** G. Henri Bogert

**Affiliations:** Paris, Ill.


### Qualifications for Parenthood.

BY DR. G. HENRI BOGERT, PARIS, ILL.

Now in the "glad Christmas time," when all the Christian world pays homage to the birth of the Child—Him who said, "Suffer little children to come"; Him whose "Inasmuch as ye have done it unto the least of these" was so new a thought—is time for us to pause and think of parenthood.

When the Little Children come, shall they come into a world of joy and well-being, equipped with the fullness of a life pulsing with possibility, or shall they come predamned by prenatal woe?

Shall the child come desired, loved from conception, its holy time in utero sweetened by love's holy bond and by a physical fitness, or shall its innocent formation be cursed by hatred, as an unavoidable by-product of unrestrained animal amorousness?

Shall it come heralded as the accidental fruit of ignorant swinishness, or shall it come as the hallowed product of intelligent love?

You may tell me that I am intruding upon the "holy of holies"; that my feet profane the most sacred relation of life.

I answer that every man and every woman have entered the priesthood of life when they have joined in that sacrament whose fruition is paternity.

When I say a sexual union that does not contemplate parenthood is bestial, I have slandered the beasts, for beasts do not descend to a level of legalized prostitution for mere physical gratification.

The priests of any cult were taught the sacred mysteries, lest they profane and desecrate the holy altar of the faith.

There is no priesthood more sacred than that which brings a new soul as its offering. There is no shrine higher than that wherein the disciples offer their own bodies as the sacrifice.

And yet, Prudery stands with frowning face and bids us pause. Prudery is the groveling superstition of today, filling the same place today in the uplift of life that she did when she drove haunting ghosts and demons and fetishes to the foreground of the altars of belief.

We inveigh bitterly against that mother whose daughter is allowed to marry until she can cook and bake and market, to be a good housewife to minister to the selfish food hunger, whose only purpose is to maintain the individual, and with the same breath we execrate that one who would teach her the true significance of the other hunger whose gratification gives new life to the world, and most of all to her who shall mother that new life.

We expend much time and effort to teach her music, that she may add gladness to those whom social intercourse brings within her circle, and we ostracise him who would teach her so that when rose petal lips shall babble the wondrous secrets of a new life to the hungering ear of motherhood, those innocent tones shall be tuned to the unison of love's best preparation.

We teach her to dress and to courtesy, that she may the better fill the amenities of daily life, and then allow her highest, holiest function, which we glorify with song and story and art as wifehood and motherhood, to bring an ill-born child to fill life's song with discords.

We take the boy who is to be her mate and devote years of his young life, at all kinds of willing sacrifice of wealth and labor, that he may meet his fellows on the plane of intelligent manhood on all the material matters of life's associations, and send him to his bride in utter ignorance of that most intimate relation known to the race. Indeed, we have allowed him to garner a training false as hell's self from the slums and from the filthiness of the same falsehood that has inculcated the double sexual standard.

We send him to the hymeneal altar as its high priest with all the gross savagery of the primal cave man accentuated by the teachings of a cryptic baseness of the one debauchery of civilization.

And from all this abyss of ignorance and falsity we expect a progeny that shall grow and progress.

We profess to wonder at the ever-growing torrent of degeneracy, insanity and disease that is pouring athwart the race.

What is conceived in corruption must be corrupt.

No fountain can flow higher than its source.

Our society, with its accepted plan of sowing wild oats as its only training school for the boy, not only adds the bestiality of the atavism of savagery in full sway to the union of sex, but in far too many cases sends him unguided to the rite of parenthood, wounded unto moral death, bankrupt in power, and poisoned with that to which the virus of asps were mercy, and yet we permit ourselves to wonder at degradation.

"Inasmuch as ye have done it unto the least of these." And yet we permit—nay, we enforce—the curse of ignorance upon these innocent pledges of creative instinct to be cursed in conception and damned ere birth.

The Preparation for Parenthood is in a full knowledge, and an adequate conception of that grand precept of the Greeks, "Know thyself." We, the social whole, have done this unto the least of these when we set up that horrible barrier of reserve and ignorance that sends the future parent all blind and helpless to the altar.

The Qualification of Parenthood most needed is a knowledge of what sex means, a conception of sexual beauty and purity and an ideal of its joyous fruition in the welcome addition of a new soul to the realm of humanity.

That knowledge will be the best safeguard of purity and continence, and yet, blind leaders of the blind, so many—oh, so many! —are clamoring for the perpetuation of this devilish reign of ignorance.

The one thing needful is a growth of the great mass mind of humanity that shall know that education is necessary, not only to the uplift of the race, but even to its preservation. The best Qualification for Parenthood is, to paraphrase Emerson, with the grandparent of the future child of purity and intelligence.

